# Institutional Questions

**Published:** 1899-12-23

**Authors:** 


### INSTITUTIONAL QUESTIONS.

### Swinging Cots on Hospital Ships.

"Interested" writes: I have noticed on the "Princess of Wales" and also on the "Maine" that the proportion of cots made to swing is very small compared to those whose position is fixed. I believe on the "Coromandel," the hospital ship fitted up at the time of the Benin Expedition, that nearly, if not quite, all the cots were swinging. Can you tell me if economy or experience is responsible for this change?

Probably a little of both. Swinging cots take more room and are possibly slightly more expensive, but it has also, we believe, been found by experience that for many cases fixed cots are preferable on board ship. The cots with which the "Princess of Wales" and the "Maine" have been fitted should answer all practical purposes. They appear to be thoroughly comfortable and well adapted to hospital use. There are a certain number of swinging cots on both ships, and if these are not found to be sufficient it would not be a very hard matter to substitute more in place of some of those now fixed.

### Select Committee on Metropolitan Hospitals.

"One of the Public" asks: Will you kindly tell me where to procure the reports of the Lords' Select Committee on Metropolitan Hospitals, which I wish to consult on one or two points of hospital administration? Also can you recommend me some book dealing with the history of the English poor law?

(1) Eyre and Spottiswoode, East Harding Street, Fleet Street, E.C., are the publishers of the Reports of the Lords' Committee. There are three of these, and the third report (price 2s. 7d.) will probably be the one to meet your requirements. You can get the books directly from the publisher or through any bookseller. (2) "A History of the English Poor Law," by Sir George Nicholls, K.C.B. (2 vols.), and a third volume by Mr. T. Mackay (P. S. King and Son, Smith Street, Westminster) would supply the information you need; and an excellent book on the poor law is Miss Twining's "Workhouses and Pauperism" (Methuen, 36, Essex Street, W.C.)

